# Data in support of the discovery of alternative splicing variants of quail LEPR and the evolutionary conservation of qLEPRl by nucleotide and amino acid sequences alignment

**DOI:** 10.1016/j.dib.2015.11.025

**Published:** 2015-11-20

**Authors:** Dandan Wang, Chunlin Xu, Taian Wang, Hong Li, Yanmin Li, Junxiao Ren, Yadong Tian, Zhuanjian Li, Yuping Jiao, Xiangtao Kang, Xiaojun Liu

**Affiliations:** a College of Animal Science and Veterinary Medicine, Henan Agricultural University, Zhengzhou 450002, China; b Henan Innovative Engineering Research Center of Poultry Germplasm Resource, Henan Agricultural University, Zhengzhou 450002, China; c International Joint Research Laboratory for Poultry Breeding of Henan, Henan Agricultural University, Zhengzhou 450002, China; d Institute of Animal Husbandry and Veterinary Medicine, Henan Academy of Agricultural Sciences, Zhengzhou 450002, China

**Keywords:** Quail LEPR, Alternative splicing, Evolutionary conservation, Sequence alignment

## Abstract

Leptin receptor (LEPR) belongs to the class I cytokine receptor superfamily which share common structural features and signal transduction pathways. Although multiple LEPR isoforms, which are derived from one gene, were identified in mammals, they were rarely found in avian except the long LEPR. Four alternative splicing variants of quail *LEPR* (*qLEPR*) had been cloned and sequenced for the first time (Wang et al., 2015 [1]). To define patterns of the four splicing variants (*qLEPRl*, *qLEPR-a*, *qLEPR-b* and *qLEPR-c*) and locate the conserved regions of *qLEPRl*, this data article provides nucleotide sequence alignment of *qLEPR* and amino acid sequence alignment of representative vertebrate LEPR. The detailed analysis was shown in [1].

**Specifications Table**

**Table:** 

Subject area	Biology
More specific subject area	Molecular function and evolution
Type of data	Figure
How data was acquired	Sequencing with sanger dideoxy sequencing
Retrieved from public databases
Data format	Analyzed
Experimental factors	Each nucleotide sequences were acquired by sequencing.
Each amino acid sequences were retrieved from NCBI or Ensemble database shown below.
Experimental features	Nucleotide and amino acid sequences were aligned by ClustalW.
Protein structure was predicted by the SMART program.
Data source location	NCBI: 〈http://www.ncbi.nlm.nih.gov/〉
Ensemble: 〈http://asia.ensembl.org/〉
SMART: 〈http://smart.embl-heidelberg.de/〉
Data accessibility	Data with this article

**Value of the data**

• qLEPR is a potentially important factor with multifunctional features, but qLEPR is completely not characterized in the literature.
• Future studies concerning effects of qLEPR in biological systems would require its characterization, which will be facilitated by the alternative splicing data in here.
• Nucleotide sequence alignment of *qLEPR* defines patterns of the splicing variants.
• Amino acid sequence alignment locates the conserved regions of LEPR in vertebrates.

## 1 Data, experimental design, materials and methods

The data shown here are two figures of sequence analysis of Japanese quail LEPR (qLEPR). Supplementary Fig. S1 is a comparison of the nucleotide sequences of four alternative splicing variants of *qLEPR* including *qLEPRl*, *qLEPR-a*, *qLEPR-b* and *qLEPR-c*. Supplementary Fig. S2 is an alignment of amino acid sequences of qLEPR with that of some other vertebrates.

### 1.1 Nucleotide sequences alignment of four *qLEPR* variants

*qLEPRl* (a long variant of *qLEPR*, GenBank: KJ639903) we first cloned was implemented a nucleotide blast in public database NCBI. The nucleotide sequence identify between *qLEPRl* and chicken *LEPR* reached 92%. Therefore, the exons and the canonical GT–AG donors and acceptor sites of *qLEPRl* were defined according to chicken genomic *LEPR* (GenBank: NC_006095). Other three variants were aligned with *qLEPRl* by ClustalW. Based on the alignment in this data, Supplementary Fig. S1 was made and its detailed analysis was shown in Ref. [1].

### 1.2 Amino acid sequences alignment of *LEPR* in vertebrates

The signal peptide and transmembrane regions of the predicated amino acid sequence of qLEPRl were detected with the SMART program (http://smart.embl-heidelberg.de/).The amino acid sequences of LEPR in other species were retrieved from public databases (including NCBI and Ensembl). The predicated amino acid sequence of qLEPRl was aligned with that of other vertebrates by ClustalW. Supplementary Fig. S2 was made according to the alignment of LEPR in vertebrates. The analysis was also shown in Ref. [1].

## Appendix A Supplementary material

Supplementary material **Supplementary Fig. 1:** A comparison of the nucleotide sequences of four alternative splicing variants of qLEPR. The qLEPRl (GenBank: KJ639903) represents a long variant; qLEPR-a (GenBank: KJ639904), qLEPR-b (GenBank: KJ639905) and qLEPR-c (GenBank: KM066117) represent other three alternative splicing variants, respectively. Exon sequences are in upper case letters, and intron sequences are in lower case letters. The canonical GT–AG donors and acceptor sites are indicated by bold letters. The dotted-lines indicate the intron sequences. The dashed-lines indicate the alternative intronic sequences. Asterisks indicate the identical coding sequences.

Supplementary material **Supplementary Fig. 2:** The amino acid sequence of quail LEPR (Coturnix japonica, GenBank: AID21692.1) was aligned with LEPR sequences of some vertebrates: Gallus gallus (GenBank: AAF31355.2), Meleagris gallopavo (GenBank: AAG40323.1), Taeniopygia guttata (GenBank: AFK25169.1), Mus musculu (GenBank: AAB95333.1), Homo sapiens (GenBank: AAB09673.1), Danio rerio (GenBank: NP_001106847.1), Anolis carolinensis (GenBank: XP_008107514.1).

## References

[1] Wang D.D., Xu C.L., Wang T.A., Li H., Li Y.M., Ren J.X., Tian Y.D., Li Z.J., Jiao Y.P., Kang X.T., Liu X.J. (2015). Discovery and functional characterization of leptin and its receptors in Japanese quail (*Coturnix japonica*). Gen. Comp. Endocrinol..

