# Supplementary material for: Data in support of the discovery of alternative splicing variants of quail LEPR and the evolutionary conservation of qLEPRl by nucleotide and amino acid sequences alignment
Source: Data Brief. 2015 Nov 20;6:1–3. doi: 10.1016/j.dib.2015.11.025 (PMC4683323; doi:10.1016/j.dib.2015.11.025)
Supplement: Supplementary file 1 — Supplementary material Supplementary Fig. 1: A comparison of the nucleotide sequences of four alternative splicing variants of qLEPR. The qLEPRl (GenBank: KJ639903) represents a long variant; qLEPR-a (GenBank: KJ639904), qLEPR-b (GenBank: KJ639905) and qLEPR-c (GenBank: KM066117) represent other three alternative splicing variants, respectively. Exon sequences are in upper case letters, and intron sequences are in lower case letters. The canonical GT–AG donors and acceptor sites are indicated by bold letters. The dotted-lines indicate the intron sequences. The dashed-lines indicate the alternative intronic sequences. Asterisks indicate the identical coding sequences. [file mmc1.doc]

649 Exon6 ←| 674

*qLEPRl* ATGGCAGTCAAGCCCATAGACATAG**gt**●●●●●●●●●●●●●●●●●●●●●●●●●●●●●●●●●●●●●●●●●

*qLEPR-a* ATGGCAGTCAAGCCCATAGACATAG**gt**●●●●**ag**GTAAGGCACATGCTGTTTAATCTGCAGTGGTTTTT

*qLEPR-b* ATGGCAGTCAAGCCCATAGACATAG**gt**●●●●●●●●●●●●●●●●●●●●●●●●●●●●●●●●●●●●●●●●●

*qLEPR-c* ATGGCAGTCAAGCCCATAGACATAG**gt**●●●●●●●●●●●●●●●●●●●●●●●●●●●●●●●●●●●●●●●●●

*************************

708 741

*qLEPRl* ●●●●●●●●●●●●●●●●●●●●●●●●●●●●●●●●●●●●●●●●●●●●●●●●●●●●●●●●●●●●●●●●●●●●

*qLEPR-a* AGCAGGATATCAGCCTTGCATGAGAGGAACTCTGCTGAATGAAGACAAGGTCAACCAAAAGGCACTAT

*qLEPR-b* ●●●●●●●●●●●●●●●●●●●●●●●●●●●●●●●●●●●●●●●●●●●●●●●●●●●●●●●●●●●●●●●●●●●●

*qLEPR-c* ●●●●●●●●●●●●●●●●●●●●●●●●●●●●●●●●●●●●●●●●●●●●●●●●●●●●●●●●●●●●●●●●●●●●

775 808 |→ Exon7

*qLEPRl* ●●●●●●●●●●●●●●●●●●●●●●●●●●●●●●●●●●●●●●●●●**ag**TAAAGCCTGAACCTCCTTTGAATGT

*qLEPR-a* ACTTACACAGTGATATGAAAAGGTCTCATTAACAGgt●●●●**ag**TAAAGCCTGAACCTCCTTTGAATGT

*qLEPR-b* ●●●●●●●●●●●●●●●●●●●●●●●●●●●●●●●●●●●●●●●●●**ag**TAAAGCCTGAACCTCCTTTGAATGT

*qLEPR-c* ●●●●●●●●●●●●●●●●●●●●●●●●●●●●●●●●●●●●●●●●●**ag**TAAAGCCTGAACCTCCTTTGAATGT

*************************

834 867

*qLEPRl* GCATCTGGAAATGACAGAGAGAGGTCAAGTGAAGATCTGCTGGTCTGAGCCTGCACCGATGCCATACC

*qLEPR-a* GCATCTGGAAATGACAGAGAGAGGTCAAGTGAAGATCTGCTGGTCTGAGCCTGCACCGATGCCATACC

*qLEPR-b* GCATCTGGAAATGACAGAGAGAGGTCAAGTGAAGATCTGCTGGTCTGAGCCTGCACCGATGCCATACC

*qLEPR-c* GCATCTGGAAATGACAGAGAGAGGTCAAGTGAAGATCTGCTGGTCTGAGCCTGCACCGATGCCATACC

********************************************************************

901 934 |→Exon8

*qLEPRl*  CCCTCCAGTATGAAGTCAACATCTCTGGAAGTTCGGGTCAAAACAGCTGGCAG**gt**●●●●**ag**GTGGCTC

*qLEPR-a* CCCTCCAGTATGAAGTCAACATCTCTGGAAGTTCGGGTCAAAACAGCTGGCAG**gt**●●●●**ag**GTGGCTC

*qLEPR-b* CCCTCCAGTATGAAGTCAACATCTCTGGAAGTTCGGGTCAAAACAGCTGGCAG**gt**●●●●**ag**GTGGCTC

*qLEPR-c* CCCTCCAGTATGAAGTCAACATCTCTGGAAGTTCGGGTCAAAACAGCTGGCAG**gt**●●●●**ag**GTGGCTC

********************************************************************

960 993

*qLEPRl* AAGTTGCTTTAAATACCTCATTAGACGTAGACAATACGCTGCTTGATTCTTCCTCCTTTGCTCAAGTG

*qLEPR-a* AAGTTGCTTTAAATACCTCATTAGACGTAGACAATACGCTGCTTGATTCTTCCTCCTTTGCTCAAGTG

*qLEPR-b* AAGTTGCTTTAAATACCTCATTAGACGTAGACAATACGCTGCTTGATTCTTCCTCCTTTGCTCAAGTG

*qLEPR-c* AAGTTGCTTTAAATACCTCATTAGACGTAGACAATACGCTGCTTGATTCTTCCTCCTTTGCTCAAGTG

********************************************************************

1027 1060

*qLEPRl* CGGTGCAAGAATCATTATGGTCCCGGGTTCTGGAGTGAGTGGAGCACGCTGTATAACCTGAATGTGGG

*qLEPR-a* CGGTGCAAGAATCATTATGGTCCCGGGTTCTGGAGTGAGTGGAGCACGCTGTATAACCTGAATGTGGG

*qLEPR-b* CGGTGCAAGAATCATTATGGTCCCGGGTTCTGGAGTGAGTGGAGCACGCTGTATAACCTGAATGTGGG

*qLEPR-c* CGGTGCAAGAATCATTATGGTCCCGGGTTCTGGAGTGAGTGGAGCACGCTGTATAACCTGAATGTGGG

********************************************************************

1094 |→Exon9 1119

*qLEPRl* AGCTGAAG**gt**●●●●**ag**TGCTGTACTTCCCTACCAAGATGCTGACCAGTGTTGGGTCTAACGTTTCATT

*qLEPR-a* AGCTGAAG**gt**●●●●**ag**TGCTGTACTTCCCTACCAAGATGCTGACCAGTGTTGGGTCTAACGTTTCATT

*qLEPR-b* AGCTGAAG**gt**●●●●**ag**TGCTGTACTTCCCTACCAAGATGCTGACCAGTGTTGGGTCTAACGTTTCATT

*qLEPR-c* AGCTGAAG**gt**●●●●**ag**TGCTGTACTTCCCTACCAAGATGCTGACCAGTGTTGGGTCTAACGTTTCATT

********************************************************************

1153 1186

*qLEPRl* TCATTGCATCTATAAAAACAAAACCAAGAGTGTAGCGTCCAAGAAGATTGTTTGGTGGCTGAACTTAG

*qLEPR-a* TCATTGCATCTATAAAAACAAAACCAAGAGTGTAGCGTCCAAGAAGATTGTTTGGTGGCTGAACTTAG

*qLEPR-b* TCATTGCATCTATAAAAACAAAACCAAGAGTGTAGCGTCCAAGAAGATTGTTTGGTGGCTGAACTTAG

*qLEPR-c* TCATTGCATCTATAAAAACAAAACCAAGAGTGTAGCGTCCAAGAAGATTGTTTGGTGGCTGAACTTAG

********************************************************************

1220 1253

*qLEPRl* CAGAAGAAATCCCAGAAAGTCAATATACGCTTGTGAACGATCGCGTAAGCAAAGTTACTCTTTTCAAC

*qLEPR-a* CAGAAGAAATCCCAGAAAGTCAATATACGCTTGTGAACGATCGCGTAAGCAAAGTTACTCTTTTCAAC

*qLEPR-b* CAGAAGAAATCCCAGAAAGTCAATATACGCTTGTGAACGATCGC**gt**----------------------

*qLEPR-c* CAGAAGAAATCCCAGAAAGTCAATATACGCTTGTGAACGATCGC**gt**----------------------

********************************************

1287 1320

*qLEPRl* TTGAAAGCAACAAAACCTAGAGGAAGTTTCTACTATAACGCGTTGTACTGTTGCCATCAAAATAGGGA

*qLEPR-a* TTGAAAGCAACAAAACCTAGAGGAAGTTTCTACTATAACGCGTTGTACTGTTGCCATCAAAATAGGGA

*qLEPR-b* --------------------------------------------------------------------

*qLEPR-c* --------------------------------------------------------------------

1354 1387 |→ Exon10

*qLEPRl* ATGTCATCATAGATACGCTGAATTATATGTAGTAG**gt**●●●●**ag**ATGTGAATATCAATATCAAATGTGA

*qLEPR-a* ATGTCATCATAGATACGCTGAATTATATGTAGTAG**gt**●●●●**ag**ATGTGAATATCAATATCAAATGTGA

*qLEPR-b* -------------------------------------●●●●**ag**ATGTGAATATCAATATCAAATGTGA

*qLEPR-c* -------------------------------------●●●●---------------------------

1413 1446

*qLEPRl* AACTGATGGGTACTTAACTAAAATGACTTGCAGATGGTCTGCAAACCCAAACGCATTGCTCTTGGGGA

*qLEPR-a* AACTGATGGGTACTTAACTAAAATGACTTGCAGATGGTCTGCAAACCCAAACGCATTGCTCTTGGGGA

*qLEPR-b* AACTGATGGGTACTTAACTAAAATGACTTGCAGATGGTCTGCAAACCCAAACGCATTGCTCTTGGGGA

*qLEPR-c* --------------------------------------------------------------------

1480 1505 |→ Exon11

*qLEPRl* GTTCCTTGCAGTTAAGATACCACAG**gt**●●●●**ag**GAGCAAAATTTATTGTTCTAACTTTCCAAGTACTC

*qLEPR-a* GTTCCTTGCAGTTAAGATACCACAG**gt**●●●●**ag**GAGCAAAATTTATTGTTCTAACTTTCCAAGTACTC

*qLEPR-b* GTTCCTTGCAGTTAAGATACCACAG**gt**●●●●**ag**GAGCAAAATTTATTGTTCTAACTTTCCAAGTACTC

*qLEPR-c* ---------------------------●●●●**ag**GAGCAAAATTTATTGTTCTAACTTTCCAAGTACTC

***********************************

1539 1594

*qLEPRl* CTCCAGAATCAGAGGTGAAAGAATGCCATTGCCAGAGGAATCATTCTTACGAGTGC

*qLEPR-a* CTCCAGAATCAGAGGTGAAAGAATGCCATTGCCAGAGGAATCATTCTTACGAGTGC

*qLEPR-b* CTCCAGAATCAGAGGTGAAAGAATGCCATTGCCAGAGGAATCATTCTTACGAGTGC

*qLEPR-c* CTCCAGAATCAGAGGTGAAAGAATGCCATTGCCAGAGGAATCATTCTTACGAGTGC

********************************************************

**Supplementary Fig. 1**: A comparison of the nucleotide sequences of four alternative splicing variants of *qLEPR.* The *qLEPRl* (GenBank: KJ639903) represents a long variant; *qLEPR-a* (GenBank: KJ639904), *qLEPR-b* (GenBank: KJ639905) and *qLEPR-c* (GenBank: KM066117) represent other three alternative splicing variants, respectively. Exon sequences are in upper case letters, and intron sequences are in lower case letters. The canonical GT-AG donors and acceptor sites are indicated by bold letters. The dotted-lines indicate the intron sequences. The dashed-lines indicate the alternative intronic sequences. Asterisks indicate the identical coding sequences.
