# Supplementary figures and images for: Data in support of the discovery of alternative splicing variants of quail LEPR and the evolutionary conservation of qLEPRl by nucleotide and amino acid sequences alignment
Source: Data Brief. 2015 Nov 20;6:1–3. doi: 10.1016/j.dib.2015.11.025 (PMC4683323; doi:10.1016/j.dib.2015.11.025)

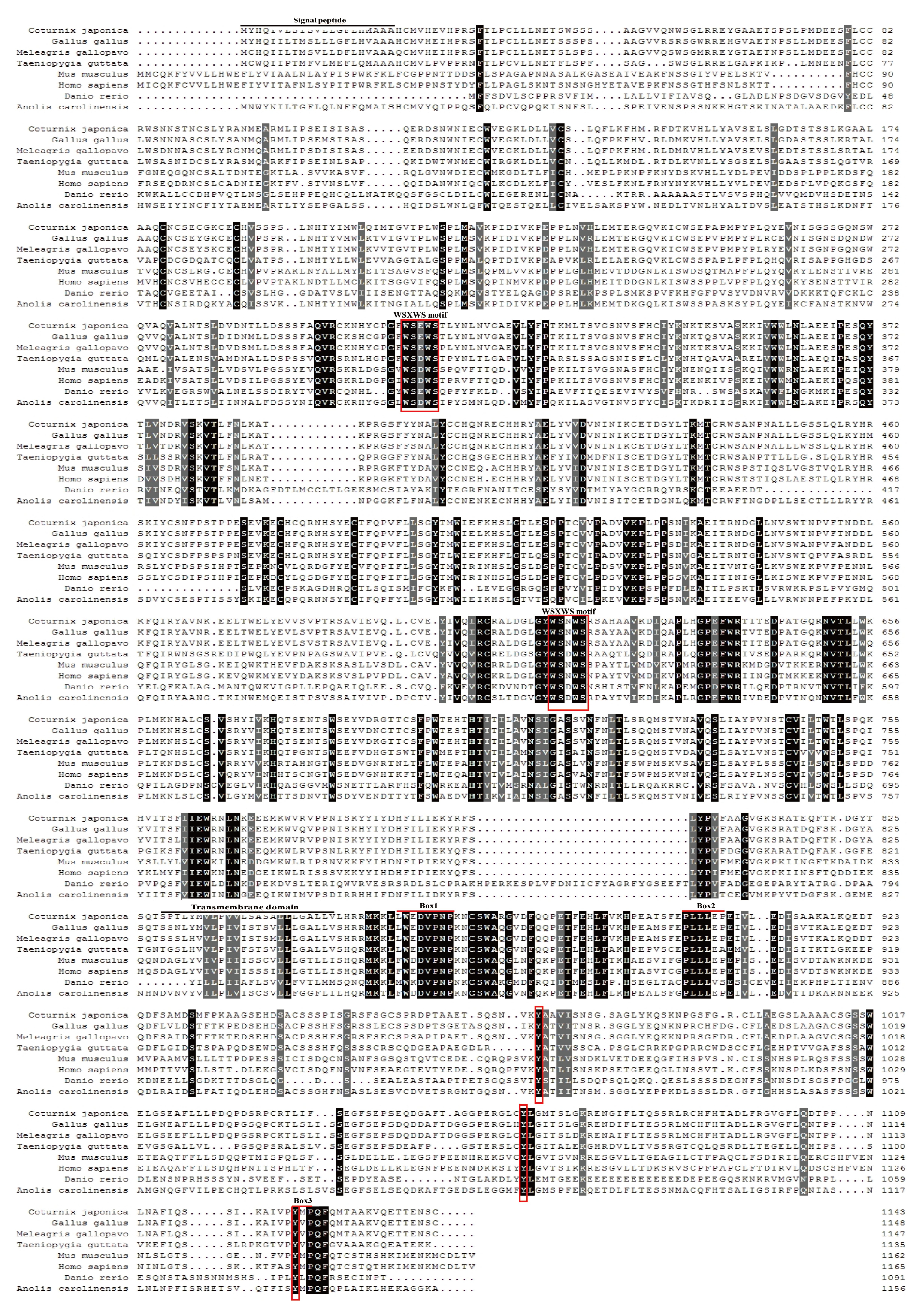

Supplement: Supplementary file 2 — Supplementary material Supplementary Fig. 2: The amino acid sequence of quail LEPR (Coturnix japonica, GenBank: AID21692.1) was aligned with LEPR sequences of some vertebrates: Gallus gallus (GenBank: AAF31355.2), Meleagris gallopavo (GenBank: AAG40323.1), Taeniopygia guttata (GenBank: AFK25169.1), Mus musculu (GenBank: AAB95333.1), Homo sapiens (GenBank: AAB09673.1), Danio rerio (GenBank: NP_001106847.1), Anolis carolinensis (GenBank: XP_008107514.1). [file mmc2.zip › mmc2.tif]
